# The Contribution of the Descending Pain Modulatory Pathway in Opioid Tolerance

**DOI:** 10.3389/fnins.2018.00886

**Published:** 2018-11-27

**Authors:** Lindsay M. Lueptow, Amanda K. Fakira, Erin N. Bobeck

**Affiliations:** ^1^Department of Psychiatry, Semel Institute for Neuroscience and Human Behavior UCLA, Los Angeles, CA, United States; ^2^Department of Pharmacological Sciences, Icahn School of Medicine at Mount Sinai, New York, NY, United States; ^3^Department of Biology, Utah State University, Logan, UT, United States

**Keywords:** opioid, tolerance, periaqueductal gray (PAG), RVM, dorsal horn

## Abstract

Opioids remain among the most effective pain-relieving therapeutics. However, their long-term use is limited due to the development of tolerance and potential for addiction. For many years, researchers have explored the underlying mechanisms that lead to this decreased effectiveness of opioids after repeated use, and numerous theories have been proposed to explain these changes. The most widely studied theories involve alterations in receptor trafficking and intracellular signaling. Other possible mechanisms include the recruitment of new structural neuronal and microglia networks. While many of these theories have been developed using molecular and cellular techniques, more recent behavioral data also supports these findings. In this review, we focus on the mechanisms that underlie tolerance within the descending pain modulatory pathway, including alterations in intracellular signaling, neural-glial interactions, and neurotransmission following opioid exposure. Developing a better understanding of the relationship between these various mechanisms, within different parts of this pathway, is vital for the identification of more efficacious, novel therapeutics to treat chronic pain.

## Descending Pain Pathway in Opioid Functions

Opioids are widely used pain therapeutics; however, the development of tolerance limits the long-term use of opioids due to the need for dose escalation over time in order to maintain analgesia. While there are four main types of opioid receptors, most pain therapeutics, including morphine, methadone, fentanyl, and oxycodone, target the mu opioid receptor (MOPr). The MOPr is a G-protein coupled receptor that couples to inhibitory heterotrimeric G-proteins (G_i/o_) producing subsequent intracellular signaling and ion conductance (Goode and Raffa, 1997; Gintzler and Chakrabarti, 2004). MOPr expression within the descending pain modulatory pathway, which includes the ventrolateral periaqueductal gray (PAG), rostral ventromedial medulla (RVM), and the dorsal horn (DH) of the spinal cord, contribute to opioid-induced antinociception and the development of opioid tolerance (Fang et al., 1989; Fairbanks and Wilcox, 1997; Tortorici et al., 2001; Morgan et al., 2006; Bobeck et al., 2009).

**FIGURE 1:**
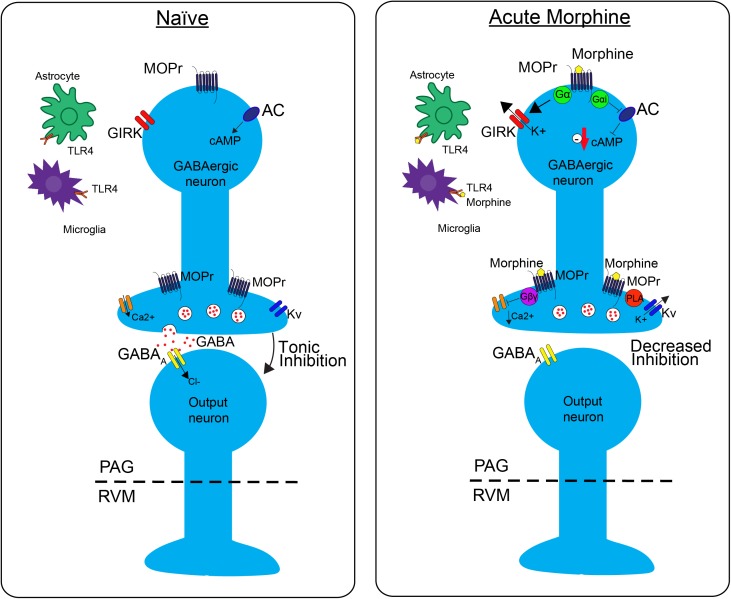
The effects of morphine on neuronal transmission in the descending pain pathway. In the **naïve state**, GABAergic interneurons in the periaqueductal gray (PAG) fire tonically, thereby producing a steady release of GABA and inhibition of PAG output neurons. Upon administration of **acute morphine**, postsynaptic mu opioid receptor (MOPr) activate GIRK channels via Gα proteins resulting in K+ release and hyperpolarization of the neuron. Additionally, MOPr activate G_i/o_ proteins, which result in the inhibition of adenylyl cyclase (AC) and decrease cAMP production. Morphine binding of presynaptic MOPr inhibits voltage dependent calcium (Ca^2+^) conductances via Gβγ proteins and activated voltage dependent potassium conductances (Kv) via Phospholipase A (PLA). Overall, these two mechanisms block release of the neurotransmitter GABA, therefore suppressing inhibition, increasing output, of the PAG neurons projecting to the rostral ventromedial medulla (RVM). Acute morphine treatment also activates toll-like receptor 4 (TLR4) receptors on astrocytes and microglia in the PAG inducing several signaling cascades.

GABAergic neurons within the PAG are a critical site of action by opioids. Under normal conditions, these neurons have tonic activity (Figure 1, naive); however, upon binding of opioids to MOPr, the activity of these neurons is decreased, disinhibiting PAG projections to the RVM (Figure 1, Acute Morphine) (Stiller et al., 1996; Vaughan et al., 1997; Bobeck et al., 2014). *In vitro* electrophysiology studies have shown that opioids reduce the frequency of spontaneous mIPSCs in PAG (Vaughan et al., 1997; Bobeck et al., 2014), which indicates a reduction in the probability of GABA release. This is also supported by *in vivo* studies. Microinjection of bicuculline (GABA_A_ agonist) into the PAG produces antinociception, which suggests that GABA is being tonically released (Bobeck et al., 2014). Furthermore, microdialysis in the PAG reveals a reduction in extracellular GABA following administration of morphine (Stiller et al., 1996).

Opioids activate different signaling cascades depending on whether the MOPr is expressed at pre- or post- synaptic sites. Opioid binding to postsynaptic MOPr results in the activation of a G-protein inwardly rectifying potassium channels (GIRK) that hyperpolarize GABAergic neurons in the PAG producing an overall decrease in GABAergic neuron activity (Figure 1; Acute Moprhine) (North and Williams, 1983; Pan et al., 1990). Alternatively, when MOPr are expressed at presynaptic sites they produce an inhibition of voltage gated calcium channels and voltage gated potassium channels (Kv) resulting in the inhibition of GABA release (Figure 1; Acute Morphine) (Wilding et al., 1995; Vaughan et al., 1997; Connor et al., 1999; Williams et al., 2001). Overall, the combined action of MOPr binding by opioids is a decrease in GABAergic neuronal activity, and therefore an increase in output from the PAG to the RVM (Figure 1; Moreau and Fields, 1986; Depaulis et al., 1987; Jacquet, 1988; Osborne et al., 1996). Recent studies support the hypothesis that this increase in PAG output to the RVM is a main contributor to the opioid-induced antinociception by demonstrating that selective inhibition of GABAergic neurons or activation of glutamatergic output neurons in the PAG mimics the antinociceptive effects of opioids (Samineni et al., 2017). In summary, these findings support the notion that analgesia is produced by disinhibition of excitatory outputs from the PAG.

The overall effect of MOPr activation in the PAG is an increase in output to the two distinct cell types within the RVM: off-cells and on-cells (Fields et al., 1983; Fields and Heinricher, 1985). The activity of off-cells pauses just prior to the response to a painful stimulus, while the activity of on-cells increases during this response, and both of these activities are blocked during the administration of opioids. There is conflicting evidence regarding the excitatory versus inhibitory nature of the PAG projections to the on- and off-cells in the RVM. Studies in GAD67-GFP mice, a marker for GABAergic neurons, show that retrogradely labeled neurons from the RVM do no co-localize with GAD67 in the PAG (Park et al., 2010), indicating that the PAG to RVM projection is glutamatergic. In contrast, studies in rats demonstrate that PAG to RVM projections are a mix of GABAergic and glutamatergic neurons (Morgan et al., 2008). Furthermore, these studies demonstrate that GABAergic neurons project from PAG and target on-cells and glutamatergic neurons project from the PAG and target off-cells (Morgan et al., 2008). Despite these differences, both studies support the notion that opioids inhibit GABA release from interneurons in the PAG, which disinhibit (i.e., excite) glutamate projections to off-cells. Given that the off-cells in the RVM are GABAergic, they subsequently inhibit pain responses in the DH (Fields et al., 1983; Moreau and Fields, 1986; Morgan et al., 2008). Overall, these studies support the concept that opioid-induced antinociception is mediated by direct excitation of off-cells and subsequent inhibition of pain in the spinal cord.

At each level of this pathway, a myriad of cellular effects drives the physiological changes mentioned above, and are highly correlated with the development of opioid tolerance. One of the most studied mechanisms involves regulation and signaling at the MOPr. Current research demonstrates that while MOPr is a key player in the development of antinociceptive tolerance, mechanisms beyond simple receptor desensitization, including alterations in neurotransmission and β-arrestin dependent signaling, are also critical. Furthermore, MOPr expression in non-neuronal cells, specifically on microglia and astrocytes within the spinal cord, and more recently within the PAG, greatly contributes to the development of opioid tolerance.

## Opioid Tolerance and Neurotransmission in the Descending Pain Pathway

Evidence suggests that tolerance is due to changes in the properties of GABAergic neurons in the PAG (Morgan et al., 2003). First of all, while microinjection of morphine into the PAG or RVM produces antinociception, repeated microinjection into the PAG and not the RVM results in tolerance (Morgan et al., 2005; Campion et al., 2016). Secondly, inhibition of MOPrs within the PAG blocks tolerance to systemic morphine (Lane et al., 2005). Furthermore, inactivation of RVM by a GABA agonist during direct administration of morphine into the PAG still leads to tolerance development (Lane et al., 2005). Therefore, MOPr within the PAG are necessary and sufficient in the development of opioid tolerance.

However, the development of tolerance within the PAG produces downstream effects along the descending pain pathway. This is evidenced by the fact that direct injection of morphine into the PAG affects RVM signaling, suggesting that their activity is in fact coupled (Tortorici et al., 2001). While acute administration of opioids into the PAG disrupts the activity of on- and off- cells in response to painful stimuli, these cells respond normally following chronic infusion that is associated with tolerance (Lane et al., 2004). Another side effect of chronic morphine treatment is hyperalgesia, or the increased sensitivity to pain following chronic morphine treatment. One theory is that hyperalgesia may manifest as opioid-induced tolerance since increased sensitivity to pain would counteract the pain-relieving effects of opioids. Some studies suggest that increased activation of the descending pain pathway by chronic morphine produces neuroadaptations with in the RVM that result in hyperalgesia (Vanderah et al., 2001). In support of this, one study demonstrated that chronic morphine produced an increase in the number of active on-cells, likely increasing sensitivity to noxious stimuli (Meng and Harasawa, 2007), which may be responsible for morphine-induced hyperalgesia. While RVM plays a role in opioid-induced tolerance, direct injections into the RVM leads to a lesser development of tolerance compared to PAG administration (Morgan et al., 2005), indicating that activation of the entire descending pain circuit is essential.

**FIGURE 2:**
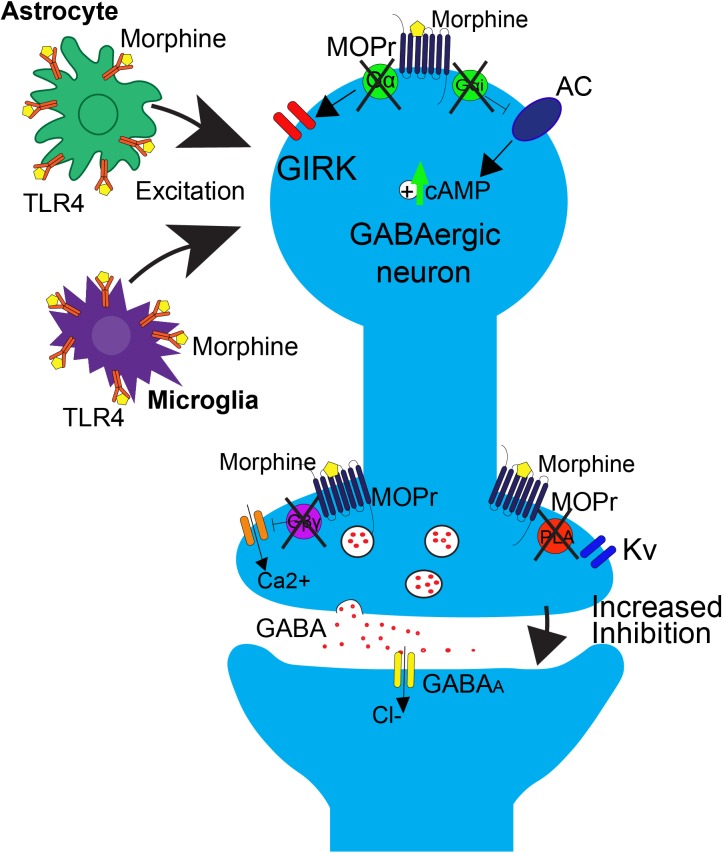
Effects of repeated morphine treatment on glial and neuronal signaling in the PAG. Chronic morphine treatment induces several side effects that block opioid-induced decreases in GABAergic interneuron activity at the neuronal level, postsynaptically. This includes the uncoupling of MOPr from G-protein mediated effects on GIRK channels and AC. This results in an upregulation of cAMP. Uncoupling also occurs in the presynaptic region, blocking the Gβγ mediated inhibition of calcium channels and PLA mediated activation of Kv channels. In this state, binding of opioids to MOPr no longer results in suppression of GABA release. At the level of glial signaling, upon repeated treatment with morphine, there is a rapid upregulation of TLR4 on astrocytes and microglia within the PAG, resulting in an increase in excitatory cytokine release, as well as a switch from G_i/o_ to G_s_ coupling at MOPr, resulting in an overall increase in excitatory tone that is correlated with opioid tolerance.

The neurophysiological mechanisms of tolerance in the PAG are mediated by MOPr uncoupling from downstream G-protein mediated signaling (Figure 2). One key study demonstrated that chronic morphine decreases opioid-mediated GIRK currents in the PAG (Bagley et al., 2005), supporting the notion that morphine tolerance is associated with uncoupling of G-protein mediated signaling. Since GIRK channels regulate neuronal excitability, this mechanism would result in a reduction in the ability of MOPr activation to suppress GABAergic neuron activity. Additionally, morphine tolerance is also associated with decreased efficacy of other MOPr agonists ability to reduce voltage gated calcium currents in the PAG (Bagley et al., 2005). The net effect of the uncoupling of MOPr activation from voltage gated calcium channels would be the attenuation of MOPr mediated inhibition of GABA release. However, the precise mechanisms underlying MOPr uncoupling from voltage gated calcium channels are complex, as cellular tolerance associated with this effect was not observed in β-arrestin two knockout mice (Connor et al., 2015), suggesting that β-arrestin two also plays a role in this interaction.

GABA release is also regulated by signaling through phospholipase A2-mediated activation of voltage gated potassium channels (Figure 1; Wimpey and Chavkin, 1991; Vaughan et al., 1997). This signaling pathway is differentially affected by morphine tolerance versus withdrawal. Morphine tolerance is associated with a decrease in opioid-mediated inhibition of GABA release (Figure 2) that is not a result of MOPr desensitization (Fyfe et al., 2010). However, during naloxone-precipitated withdrawal following chronic morphine, GABA release is enhanced via an increase in adenylyl cyclase (AC) signaling (Sharma et al., 1975; Ingram et al., 1998; Hack et al., 2003). These two outcomes may be related as studies have demonstrated that inhibition of AC in the PAG prevents the development and expression of morphine tolerance (Bobeck et al., 2014). Moreover, AC activation is sufficient to increase GABA release from PAG neurons (Bobeck et al., 2014).

## Intracellular Signaling Changes in the PAG-RVM-DH Pathway in Opioid Tolerance

Direct activation of the MOPr results in the Gα subunit-mediated inhibition of the AC-cyclic adenosine monophosphate (cAMP)-protein kinase A (PKA) pathway (Figure 1; Sharma et al., 1975; Guitart and Nestler, 1989; Hirst and Lambert, 1995). However, opioid binding activates other signaling proteins, such as protein kinase C (PKC) and extracellular signal-regulated kinase 1/2 (ERK1/2) via β-arrestin pathways, which are independent of G-protein signaling. As mentioned previously, downstream of G-protein mediated signaling, there is an inhibition of calcium channels and activation of potassium channels that leads to hyperpolarization and a reduction in neurotransmitter release in the PAG that produces antinociception (Bourinet et al., 1996; Ippolito et al., 2002; Torrecilla et al., 2002). Chronic morphine produces adaptations that contribute to opioid-tolerance within all these downstream signaling pathways.

Long-term opioid treatment leads to adaptations in many signaling proteins within the PAG-RVM-DH pathway, which have been proposed as mechanisms of opioid tolerance. In contrast to the acute inhibitory effect of opioids on cAMP production, chronic morphine treatment upregulates cAMP (Figure 2; Guitart and Nestler, 1989; Gintzler and Chakrabarti, 2004). It has been proposed that this upregulation in cAMP is caused by compensatory changes in intracellular signaling, or an uncoupling of G_i/o_-proteins from the receptor and a switch to coupling with G_s_-proteins (Gintzler and Chakrabarti, 2004). Very few *in vivo* studies have evaluated the role of the AC pathway in morphine tolerance, but inhibition of the AC pathway, via either intracerebroventricular (ICV) or intra-PAG injection, during morphine pretreatment has been shown to block the development of morphine tolerance (Smith et al., 2006; Gabra et al., 2008; Bobeck et al., 2014). In the DH, administration of morphine results in no change or even an increase in MOPr expression, but a significant down-regulation of the G-protein activation in the DH, as measured by [^35^S]-GTPyS (Maher et al., 2001; Ray et al., 2004). The loss of G-protein signaling is likely a switch in MOPr G-protein coupling, from G_i/o_ to G_s_ coupling (Gintzler and Chakrabarti, 2004). Recently, adrenomedullin, a pronociceptive peptide from the CGRP family, has been implicated in mediating this G-protein switch in the DH. Following chronic morphine, adrenomedullin is significantly upregulated in the DH and dorsal root ganglia, and inhibition of its receptor prevents or reverses morphine tolerance and blocks the MOPr G_i/o_ to G_s_ switch in coupling (Wang et al., 2016).

Behavioral studies suggest that the mechanisms underlying tolerance are dependent on the specific MOPr agonist being studied. Some agonists, such as morphine, do not readily recruit β-arrestin or internalize the receptor, as compared to high efficacy agonists, such as DAMGO or fentanyl, which readily do both. This difference in signaling suggests differences in tolerance mechanisms, where morphine-mediated tolerance utilizes a G-protein dependent mechanism, and other MOPr agonists, such as DAMGO or fentanyl, use a β-arrestin dependent mechanism (Hull et al., 2010; Melief et al., 2010; Bobeck et al., 2014, 2016; Morgan et al., 2014). For example, inhibition of ERK1/2 within the PAG during the development of tolerance enhances morphine tolerance (Macey et al., 2009), but reduces tolerance to DAMGO and has no effect on fentanyl tolerance (Bobeck et al., 2016). Furthermore, inhibition of the G-protein dependent pathway (i.e., c-Jun N-terminal kinase) blocks development of tolerance to morphine, but not fentanyl. However, inhibition of β-arrestin dependent signaling (i.e., G protein-coupled receptor kinase) blocks expression of fentanyl tolerance (Morgan et al., 2014).

Neuropeptides within the descending pain pathway have also been shown to regulate opioid tolerance. One such neuropeptide, cholecystokinin (CCK), is particularly enriched in supraspinal midbrain regions known to regulate spinal nociception (King et al., 2005). There is evidence that CCK acting within the PAG-RVM-DH pathway regulates morphine tolerance (Xie et al., 2005; Thomas et al., 2015). A CCK receptor antagonist, directly injected into the PAG, is able to block morphine tolerance (Xie et al., 2005). In the RVM, injection of CCK blocks opioid activation of off-cells that mediate descending antinociception, resulting in a blockade of the analgesic effects of morphine (Xu et al., 2014; Thomas et al., 2015).

*N*-methyl D-aspartate receptors (NMDArs) have been heavily implicated in the development of both spinal-mediated hyperalgesia and opioid tolerance. NMDAr antagonists or the targeted disruption of the NR2 subunits, NR2a and NR2b, attenuates opioid tolerance (Price et al., 2000; Zhao et al., 2012). Deletion of PSD-93, the anchoring protein for NR2a and NR2b in the synapse, leads to a DH site-specific down-regulation of both subunits from the plasma membrane into the cytosolic compartment, and a reduction in the development of morphine tolerance (Liaw et al., 2008). This is a region-specific effect, as other portions of the descending pain pathway did not see changes in the NR2 subunit localization (Kozela and Popik, 2007). Interestingly, NMDArs in the PAG have not been implicated in tolerance (Morgan et al., 2009).

A few other main signaling targets have been implicated in DH-mediated opioid tolerance, as well. Mammalian target of rapamycin (mTOR) is found to be upregulated following repeated intrathecal morphine administration, and this effect is mediated by activation of PI3K/AKT following MOPr activation (Xu et al., 2015). Administration of mTOR inhibitors is able to both prevent and reverse morphine tolerance (Xu et al., 2014, 2015; Jiang et al., 2016; Chen et al., 2017). Calcium/calmodulin-dependent protein kinase IIα has also been implicated in the development of tolerance (Brüggemann et al., 2000). It has been shown to colocalize with MOPr, in the DH specifically, following opioid administration, possibly resulting in increased MOPr phosphorylation and desensitization (Brüggemann et al., 2000).

## Impact of Opioid-Induced Neuroinflammation on the Development of Tolerance

Over the past few decades, researchers have discovered that opioids are potent activators of immune cells within the CNS, and this inflammation is a strong contributor to the development of opioid tolerance (Giron et al., 2015; Cahill and Taylor, 2017). Specifically, repeated administration of opioids, which leads to activation of glia within the PAG and spinal cord of the descending pain pathway, results in alterations in both intracellular signaling cascades and signaling properties of neurons. Furthermore, microglial inhibitors have been shown to attenuate morphine-induced tolerance (Song and Zhao, 2001; Raghavendra et al., 2002, 2004; Cui et al., 2008; Eidson and Murphy, 2013; Harada et al., 2013). Though the precise mechanisms that underlie these changes are only beginning to be uncovered, a few notable pathways have emerged that are likely significant contributors to the development of opioid tolerance.

One prominent pro-inflammatory signaling cascade that has been implicated in opioid tolerance involves the immune receptor, toll-like receptor 4 (TLR4, Figure 2). Upon agonist binding to TRL4, sphingomyelinase produces ceramide, which allows for interaction of the receptor with its co-activators myeloid differentiation factor-2 (MD-2) and CD14, resulting in subsequent activation of 3 parallel pathways: the p38-MAPK pathway, the PI3K/AKT pathway (cell survival/apoptosis), and the NFκB pathway (proinflammatory cytokine release) (Rönnbäck and Hansson, 1988; Watkins et al., 2009; Nakamoto et al., 2012; Eidson and Murphy, 2013). In the spinal cord, TLR4 is primarily expressed on microglial cells and is shown to be upregulated (Figure 2) along with its cofactor MD-2 following morphine treatment (Wang et al., 2012), and activation of TLR4 signaling can induce “naïve tolerance” to opioids (Eidson and Murphy, 2013; Grace et al., 2015). Furthermore, inhibition of TLR4, co-activators MD-2 or CD14, or inhibition of ceramide biosynthesis, leads to attenuation of morphine tolerance, as well as decreased microglial activation, suggesting a prominent role for the TLR4 pathway in the development of opioid tolerance, at the level of the spinal cord (Ndengele et al., 2009; Hutchinson et al., 2010, 2011; Muscoli et al., 2010; Thomas et al., 2015).

Interestingly, it is also thought that TLR4 is directly activated by opioids (Figures 1, 2; Hutchinson et al., 2011; Wang et al., 2012; Grace et al., 2015), and, perhaps more importantly, the accessory protein MD-2 is able to non-stereoselectively bind opioids and signal through TRL4 (Grace et al., 2015). Since the classic opioid receptors only bind the (−)-opioid isomer, the (+)-opioid isomer antagonists could be used to block TLR4-mediated microglial activation and pro-inflammatory cytokine production. In fact, studies have demonstrated that (+)-naloxone is able to attenuate morphine-induced analgesia, specifically at the level of the spinal cord (Hutchinson et al., 2010; Lewis et al., 2010). This non-stereoselectivity at the TLR4 receptor complex could potentially be leveraged for the enhancement of the therapeutic efficacy of opioids, including enhancing analgesic effects and reducing tolerance.

How does the activation of glial cells lead to alterations in neuronal signaling? One possibility is through the alteration of neuronal excitability via increased release of glially-derived pro-inflammatory cytokines, including TNF (tumor necrosis factor) and IL-1β, which are known to increase neuronal AMPA and NMDA receptors, as well as down regulate GABA receptors (Viviani et al., 2003; Stellwagen, 2005). Within the PAG, repeated morphine administration results in an upregulation of TLR4, which subsequently leads to an increase in release of TNF and IL-1β (Eidson and Murphy, 2013; Eidson et al., 2017). This upregulation is concurrent with a downregulation of astrocyte glutamate transporters GLT-1 and GLAST, which are responsible for synaptic glutamate uptake. The overall effect is an increase in neuronal excitability, thereby lowering the ability of opioids to hyperpolarize mu-containing GABAergic neurons (Figure 2). Within the PAG to RVM circuitry, this results in an inability for morphine to disinhibit output neurons to RVM (Eidson and Murphy, 2013; Eidson et al., 2017).

Another potential point of cross talk is via purinergic receptors, specifically P2X4 and P2X7, which are primarily expressed on microglia. These receptors are also capable of upregulating pro-inflammatory cytokines, and blocking their activity in the spinal cord attenuates morphine tolerance (Horvath et al., 2010; Zhou et al., 2010; Xiao et al., 2015). P2X4 activates the p38-MAPK pathway, resulting in the release of IL-1β, TNF-α, and BDNF, which, as mentioned above, are known to alter neuronal excitability and contribute to pain hypersensitivity, but no direct connection has been made to opioid tolerance (Ferrini et al., 2013; Grace et al., 2015; Thomas et al., 2015). However, P2X7 mediated release of IL-18 from microglia induces activation of the IL-18 receptor on astrocytes, thereby increasing the release of D-serine, which is able to activate NMDA receptors in neurons. Activation of both receptors is able to alter glial activation and neuronal excitability, suggesting a complicated crosstalk between cell types in the spinal cord that is correlated with morphine tolerance (Chen et al., 2012).

## Conclusion

The descending pain pathway is a critical modulator of nociception and plays an important role in mediating endogenous and exogenous opioid-induced analgesia. Because of this, it is highly implicated in allostatic cellular and molecular changes following repeated opioid use that lead to the development of tolerance. While this review has touched on a number of those changes at each level of the descending pain pathway, including desensitization of MOPr, altered cellular excitability and signaling, and induction of immune-competent cells, we do not yet have a complete understanding of all the factors that might be contributing to opioid tolerance.

Much of the literature on opioid tolerance has focused the effects of morphine on this system. Future research must expand to include other commonly used opioids, especially in light of the increasing use of oxycodone and fentanyl, as each of these has widely different pharmacokinetic and signaling profiles, and may have differential effects on each level of the PAG-RVM-DH pathway. Indeed, studies looking at cross-tolerance between opioid analgesics suggest that differences in the distribution of the drug within the pain pathway may be differentially contributing to the development of tolerance. Furthermore, the cellular signaling pathways initiated within these spinal and supraspinal regions following administration of different opioids are known to vary.

Finally, the research design of the studies related to opioids and tolerance has varied widely in terms of not only the drugs used, but also routes of administration, length of exposure, and use of biological systems. Also, the majority of studies on opioid tolerance have focused on males and have largely excluded females. Given that males show greater morphine potency, tolerance, and activation of neurons from PAG to RVM following morphine, as compared to females (Loyd et al., 2008), it is imperative to further explore these differences. Overall, these variations in research design have resulted in a myriad of observed cellular changes that correlate with tolerance, but with no definite conclusions or unifying theories of tolerance. While no one specific etiology may exist, future researchers must be careful in designing these studies, in order to make meaningful conclusions regarding the cellular impact of opioids in the development of tolerance.

## Author Contributions

LL, AF, and EB contributed to the writing and editing of this manuscript.

## Conflict of Interest Statement

The authors declare that the research was conducted in the absence of any commercial or financial relationships that could be construed as a potential conflict of interest.

## References

[B1] Bagley E. E. Chieng B. C. H. Christie M. J. Connor M. (2005). Opioid tolerance in periaqueductal gray neurons isolated from mice chronically treated with morphine. *Br. J. Pharmacol.* 146 68–76. 10.1038/sj.bjp.0706315 15980868 PMC1576256

[B2] Bobeck E. N. Chen Q. Morgan M. M. Ingram S. L. (2014). Contribution of adenylyl cyclase modulation of pre- and postsynaptic GABA neurotransmission to morphine antinociception and tolerance. *Neuropsychopharmacology* 39 2142–2152. 10.1038/npp.2014.62 24622471 PMC4104331

[B3] Bobeck E. N. Ingram S. L. Hermes S. M. Aicher S. A. Morgan M. M. (2016). Ligand-biased activation of extracellular signal-regulated kinase 1/2 leads to differences in opioid induced antinociception and tolerance. *Behav. Brain Res.* 298 17–24. 10.1016/j.bbr.2015.10.032 26497105 PMC4779316

[B4] Bobeck E. N. McNeal A. L. Morgan M. M. (2009). Drug dependent sex-differences in periaqueducatal gray mediated antinociception in the rat. *Pain* 147 210–216. 10.1016/j.pain.2009.09.008 19796879 PMC2814455

[B5] Bourinet E. Soong T. W. Stea A. Snutch T. P. (1996). Determinants of the G protein-dependent opioid modulation of neuronal calcium channels. *Proc. Natl. Acad. Sci. U.S.A.* 93 1486–1491. 10.1073/pnas.93.4.1486 8643659 PMC39966

[B6] Brüggemann I. Schulz S. Wiborny D. Höllt V. (2000). Colocalization of the μ-opioid receptor and calcium/calmodulin-dependent kinase II in distinct pain-processing brain regions. *Mol. Brain Res.* 85 239–250. 10.1016/S0169-328X(00)00265-5 11146127

[B7] Cahill C. M. Taylor A. M. (2017). Neuroinflammation—a co-occurring phenomenon linking chronic pain and opioid dependence. *Curr. Opin. Behav. Sci.* 13 171–177. 10.1016/j.cobeha.2016.12.003 28451629 PMC5404697

[B8] Campion K. N. Saville K. A. Morgan M. M. (2016). Relative contribution of the dorsal raphe nucleus and ventrolateral periaqueductal gray to morphine antinociception and tolerance in the rat. *Eur. J. Neurosci.* 44 2667–2672. 10.1111/ejn.13378 27564986 PMC5300757

[B9] Chen M. L. Cao H. Chu Y. X. Cheng L. Z. Liang L. L. Zhang Y. Q. (2012). Role of P2X7 receptor-mediated IL-18/IL-18R signaling in morphine tolerance: Multiple glial-neuronal dialogues in the rat spinal cord. *J. Pain* 13 945–958. 10.1016/j.jpain.2012.06.007 22968128

[B10] Chen S.-P. Zhou Y.-Q. Liu D.-Q. Zhang W. Manyande A. Guan X.-H. (2017). PI3K/Akt pathway: a potential therapeutic target for chronic pain. *Curr. Pharm. Des.* 23 1860–1868. 10.2174/1381612823666170210150147 28190392

[B11] Connor M. Bagley E. E. Chieng B. C. Christie M. J. (2015). β-Arrestin-2 knockout prevents development of cellular μ-opioid receptor tolerance but does not affect opioid-withdrawal-related adaptations in single PAG neurons. *Br. J. Pharmacol.* 172 492–500. 10.1111/bph.12673 24597632 PMC4292963

[B12] Connor M. Schuller A. Pintar J. E. Christie M. J. (1999). μ-opioid receptor modulation of calcium channel current in periaqueductal grey neurons from C57B16/J mice and mutant mice lacking MOR-1. *Br. J. Pharmacol.* 126 1553–1558. 10.1038/sj.bjp.0702457 10323586 PMC1565931

[B13] Cui Y. Liao X. X. Liu W. Guo R. X. Wu Z. Z. Zhao C. M. (2008). A novel role of minocycline: attenuating morphine antinociceptive tolerance by inhibition of p38 MAPK in the activated spinal microglia. *Brain. Behav. Immun.* 22 114–123. 10.1016/j.bbi.2007.07.014 17919885

[B14] Depaulis A. Morgan M. M. Liebeskind J. C. (1987). GABAergic modulation of the analgesic effects of morphine microinjected in the ventral periaqueductal gray matter of the rat. *Brain Res.* 436 223–228. 10.1016/0006-8993(87)91665-9 3435824

[B15] Eidson L. N. Inoue K. Young L. J. Tansey M. G. Murphy A. Z. (2017). Toll-like receptor 4 mediates morphine-induced neuroinflammation and tolerance via soluble tumor necrosis factor signaling. *Neuropsychopharmacology* 42 661–670. 10.1038/npp.2016.131 27461080 PMC5240168

[B16] Eidson L. N. Murphy A. Z. (2013). Blockade of toll-like receptor 4 attenuates morphine tolerance and facilitates the pain relieving properties of morphine. *J. Neurosci.* 33 15952–15963. 10.1523/JNEUROSCI.1609-13.2013 24089500 PMC3787504

[B17] Fairbanks C. A. Wilcox G. L. (1997). Acute tolerance to spinally administered morphine compares mechanistically with chronically induced morphine tolerance. *J. Pharmacol. Exp. Ther.* 282 1408–1417. 9316854

[B18] Fang F. G. Haws C. M. Drasner K. Williamson A. Fields H. L. (1989). Opioid peptides (DAGO-enkephalin, dynorphin A(1-13), BAM 22P) microinjected into the rat brainstem: comparison of their antinociceptive effect and their effect on neuronal firing in the rostral ventromedial medulla. *Brain Res.* 501 116–128. 10.1016/0006-8993(89)91033-0 2572306

[B19] Ferrini F. Trang T. Mattioli T. A. M. Laffray S. Del’Guidice T. Lorenzo L. E. (2013). Morphine hyperalgesia gated through microglia-mediated disruption of neuronal Cl-homeostasis. *Nat. Neurosci.* 16 183–192. 10.1038/nn.3295 23292683 PMC4974077

[B20] Fields H. L. Bry J. Hentall I. Zorman G. (1983). The activity of neurons in the rostral medulla of the rat during withdrawal from noxious heat. *J. Neurosci.* 3 2545–2552. 10.1523/JNEUROSCI.03-12-02545.1983 6317812 PMC6564660

[B21] Fields H. L. Heinricher M. M. (1985). Anatomy and physiology of a nociceptive modulatory system. *Philos. Trans. R. Soc. Lond. B. Biol. Sci.* 308 361–374. 10.1098/rstb.1985.0037 2858889

[B22] Fyfe L. W. Cleary D. R. Macey T. A. Morgan M. M. Ingram S. L. (2010). Tolerance to the antinociceptive effect of morphine in the absence of short-term presynaptic desensitization in rat periaqueductal gray neurons. *J. Pharmacol. Exp. Ther.* 335 674–680. 10.1124/jpet.110.172643 20739455 PMC2993552

[B23] Gabra B. H. Bailey C. P. Kelly E. Smith F. L. Henderson G. Dewey W. L. (2008). Pre-treatment with a PKC or PKA inhibitor prevents the development of morphine tolerance but not physical dependence in mice. *Brain Res.* 1217 70–77. 10.1016/j.brainres.2008.04.036 18501877 PMC3773693

[B24] Gintzler A. R. Chakrabarti S. (2004). Chronic morphine-induced plasticity among signalling molecules. *Novartis Found. Symp.* 261 191–193. 10.1002/0470869127.ch13 15469050

[B25] Giron S. E. Griffis C. A. Burkard J. F. (2015). Chronic pain and decreased opioid efficacy: an inflammatory link. *Pain Manag. Nurs.* 16 819–831. 10.1016/j.pmn.2015.04.001 25962543

[B26] Goode T. L. Raffa R. B. (1997). An examination of the relationship between mu-opioid antinociceptive efficacy and G-protein coupling using pertussis and cholera toxins. *Life Sci.* 60 l107–l113. 10.1016/S0024-3205(96)00684-4 9042382

[B27] Grace P. M. Maier S. F. Watkins L. R. (2015). Opioid-Induced central immune signaling: implications for opioid analgesia. *Headache* 55 475–489. 10.1111/head.12552 25833219 PMC4402135

[B28] Guitart X. Nestler E. J. (1989). Identification of morphine- and cyclic AMP-regulated phosphoproteins (MARPPs) in the locus coeruleus and other regions of rat brain: regulation by acute and chronic morphine. *J. Neurosci.* 9 4371–4387. 10.1523/JNEUROSCI.09-12-04371.1989 2556507 PMC6569648

[B29] Hack S. P. Vaughan C. W. Christie M. J. (2003). Modulation of GABA release during morphine withdrawal in midbrain neurons in vitro. *Neuropharmacology* 45 575–584. 10.1016/S0028-3908(03)00205-3 12941371

[B30] Harada S. Nakamoto K. Tokuyama S. (2013). The involvement of midbrain astrocyte in the development of morphine tolerance. *Life Sci.* 93 573–578. 10.1016/j.lfs.2013.08.009 23988850

[B31] Hirst R. A. Lambert D. G. (1995). Adenylyl cyclase in SH-SY5Y human neuroblastoma cells is regulated by intra- and extracellular calcium. *Biochem. Pharmacol.* 49 1633–1640. 10.1016/0006-2952(95)00075-B 7786304

[B32] Horvath R. J. Romero-Sandoval E. A. Leo J. A. D. (2010). Inhibition of microglial P2X4receptors attenuates morphine tolerance, Iba1, GFAP and μ opioid receptor protein expression while enhancing perivascular microglial ED2. *Pain* 150 401–413. 10.1016/j.pain.2010.02.042 20573450 PMC2921455

[B33] Hull L. C. Llorente J. Gabra B. H. Smith F. L. Kelly E. Bailey C. (2010). The effect of protein kinase C and G protein-coupled receptor kinase inhibition on tolerance induced by mu-opioid agonists of different efficacy. *J. Pharmacol. Exp. Ther.* 332 1127–1135. 10.1124/jpet.109.161455 20008489 PMC2835442

[B34] Hutchinson M. R. Shavit Y. Grace P. M. Rice K. C. Maier S. F. Watkins L. R. (2011). Exploring the neuroimmunopharmacology of opioids: an integrative review of mechanisms of central immune signaling and their implications for opioid analgesia. *Pharmacol. Rev.* 63 772–810. 10.1124/pr.110.004135 21752874 PMC3141878

[B35] Hutchinson M. R. Zhang Y. Shridhar M. Evans J. H. Buchman M. M. Zhao T. X. (2010). Evidence that opioids may have toll-like receptor 4 and MD-2 effects. *Brain Behav. Immun.* 24 83–95. 10.1016/j.bbi.2009.08.004 19679181 PMC2788078

[B36] Ingram S. L. Vaughan C. W. Bagley E. E. Connor M. Christie M. J. (1998). Enhanced opioid efficacy in opioid dependence is caused by an altered signal transduction pathway. *J. Neurosci.* 18 10269–10276. 10.1523/JNEUROSCI.18-24-10269.1998 9852564 PMC6793345

[B37] Ippolito D. L. Temkin P. A. Rogalski S. L. Chavkin C. (2002). N-terminal tyrosine residues within the potassium channel Kir3 modulate GTPase activity of Galphai. *J. Biol. Chem.* 277 32692–32696. 10.1074/jbc.M204407200 12082117 PMC1414899

[B38] Jacquet Y. F. (1988). The NMDA receptor: central role in pain inhibition in rat periaqueductal gray. *Eur. J. Pharmacol.* 154 271–276. 10.1016/0014-2999(88)90201-4 2853058

[B39] Jiang Z. Wu S. Wu X. Zhong J. Lv A. Jiao J. (2016). Blocking mammalian target of rapamycin alleviates bone cancer pain and morphine tolerance via μ-opioid receptor. *Int. J. Cancer* 138 2013–2020. 10.1002/ijc.29927 26566757

[B40] King T. Ossipov M. H. Vanderah T. W. Porreca F. Lai J. (2005). Is paradoxical pain induced by sustained opioid exposure an underlying mechanism of opioid antinociceptive tolerance? *Neurosignals* 14 194–205. 10.1159/000087658 16215302

[B41] Kozela E. Popik P. (2007). A complete analysis of NMDA receptor subunits in periaqueductal grey and ventromedial medulla of morphine tolerant mice. *Drug Alcohol Depend.* 86 290–293. 10.1016/j.drugalcdep.2006.06.018 16930867

[B42] Lane D. A. Patel P. A. Morgan M. M. (2005). Evidence for an intrinsic mechanism of antinociceptive tolerance within the ventrolateral periaqueductal gray of rats. *Neuroscience* 135 227–234. 10.1016/J.NEUROSCIENCE.2005.06.014 16084660

[B43] Lane D. A. Tortorici V. Morgan M. M. (2004). Behavioral and electrophysiological evidence for tolerance to continuous morphine administration into the ventrolateral periaqueductal gray. *Neuroscience* 125 63–69. 10.1016/j.neuroscience.2004.01.023 15051146

[B44] Lewis S. S. Hutchinson M. R. Rezvani N. Loram L. C. Zhang Y. Maier S. F. (2010). Evidence that intrathecal morphine-3-glucuronide may cause pain enhancement via toll-like receptor 4/MD-2 and interleukin-1β. *Neuroscience* 165 569–583. 10.1016/j.neuroscience.2009.10.011 19833175 PMC2795035

[B45] Liaw W. J. Zhu X. G. Yaster M. Johns R. A. Gauda E. B. Tao Y. X. (2008). Distinct expression of synaptic NR2A and NR2B in the central nervous system and impaired morphine tolerance and physical dependence in mice deficient in postsynaptic density-93 protein. *Mol. Pain* 4:45. 10.1186/1744-8069-4-45 18851757 PMC2576175

[B46] Loyd D. R. Morgan M. M. Murphy A. Z. (2008). Sexually dimorphic activation of the periaqueductal gray-rostral ventromedial medullary circuit during the development of tolerance to morphine in the rat. *Eur. J. Neurosci.* 27 1517–1524. 10.1111/j.1460-9568.2008.06100.x 18364026 PMC2821209

[B47] Macey T. A. Bobeck E. N. Hegarty D. M. Aicher S. A. Ingram S. L. Morgan M. M. (2009). Extracellular signal-regulated kinase 1/2 activation counteracts morphine tolerance in the periaqueductal gray of the rat. *J. Pharmacol. Exp. Ther.* 331 412–418. 10.1124/jpet.109.152157 19684256 PMC2775267

[B48] Maher C. E. Eisenach J. C. Pan H. L. Xiao R. Childers S. R. (2001). Chronic intrathecal morphine administration produces homologous mu receptor/G-protein desensitization specifically in spinal cord. *Brain Res.* 895 1–8. 10.1016/S0006-8993(00)03093-6 11259753

[B49] Melief E. J. Miyatake M. Bruchas M. R. Chavkin C. (2010). Ligand-directed c-Jun N-terminal kinase activation disrupts opioid receptor signaling. *Proc. Natl. Acad. Sci. U.S.A.* 107 11608–11613. 10.1073/pnas.1000751107 20534436 PMC2895055

[B50] Meng I. D. Harasawa I. (2007). Chronic morphine exposure increases the proportion of on-cells in the rostral ventromedial medulla in rats. *Life Sci.* 80 1915–1920. 10.1016/j.lfs.2007.02.022 17400254 PMC2736558

[B51] Moreau J. L. Fields H. L. (1986). Evidence for GABA involvement in midbrain control of medullary neurons that modulate nociceptive transmission. *Brain Res.* 397 37–46. 10.1016/0006-8993(86)91367-3 3801864

[B52] Morgan M. M. Bobeck E. N. Ingram S. L. (2009). Glutamate modulation of antinociception, but not tolerance, produced by morphine microinjection into the periaqueductal gray of the rat. *Brain Res.* 1295 59–66. 10.1016/j.brainres.2009.07.100 19664608 PMC2772196

[B53] Morgan M. M. Clayton C. C. Boyer-Quick J. S. (2005). Differential susceptibility of the PAG and RVM to tolerance to the antinociceptive effect of morphine in the rat. *Pain* 113 91–98. 10.1016/j.pain.2004.09.039 15621368

[B54] Morgan M. M. Clayton C. C. Lane D. A. (2003). Behavioral evidence linking opioid-sensitive GABAergic neurons in the ventrolateral periaqueductal gray to morphine tolerance. *Neuroscience* 118 227–232. 10.1016/S0306-4522(02)00822-9 12676152

[B55] Morgan M. M. Fossum E. N. Levine C. S. Ingram S. L. (2006). Antinociceptive tolerance revealed by cumulative intracranial microinjections of morphine into the periaqueductal gray in the rat. *Pharmacol. Biochem. Behav.* 85 214–219. 10.1016/j.pbb.2006.08.003 16979226

[B56] Morgan M. M. Reid R. A. Saville K. A. (2014). Functionally selective signaling for morphine and fentanyl antinociception and tolerance mediated by the rat periaqueductal gray. *PLoS One* 9:e114269. 10.1371/journal.pone.0114269 25503060 PMC4263532

[B57] Morgan M. M. Whittier K. L. Hegarty D. M. Aicher S. A. (2008). Periaqueductal gray neurons project to spinally projecting GABAergic neurons in the rostral ventromedial medulla. *Pain* 140 376–386. 10.1016/j.pain.2008.09.009 18926635 PMC2704017

[B58] Muscoli C. Doyle T. Dagostino C. Bryant L. Chen Z. Watkins L. R. (2010). Counter-regulation of opioid analgesia by glial-derived bioactive sphingolipids. *J. Neurosci.* 30 15400–15408. 10.1523/JNEUROSCI.2391-10.2010 21084596 PMC3000610

[B59] Nakamoto K. Kawasaki S. Kobori T. Fujita-Hamabe W. Mizoguchi H. Yamada K. (2012). Involvement of matrix metalloproteinase-9 in the development of morphine tolerance. *Eur. J. Pharmacol.* 683 86–92. 10.1016/j.ejphar.2012.03.006 22445883

[B60] Ndengele M. M. Cuzzocrea S. Masini E. Vinci M. C. Esposito E. Muscoli C. (2009). Spinal ceramide modulates the development of morphine antinociceptive tolerance via peroxynitrite-mediated nitroxidative stress and neuroimmune activation. *J. Pharmacol. Exp. Ther.* 329 64–75. 10.1124/jpet.108.146290 19033555 PMC2670603

[B61] North R. A. Williams J. T. (1983). Opiate activation of potassium conductance inhibits calcium action potentials in rat locus coeruleus neurones. *Br. J. Pharmacol.* 80 225–228. 10.1111/j.1476-5381.1983.tb10023.x 6652378 PMC2045013

[B62] Osborne P. B. Vaughan C. W. Wilson H. I. Christie M. J. (1996). Opioid inhibition of rat periaqueductal grey neurones with identified projections to rostral ventromedial medulla in vitro. *J. Physiol.* 490 383–389. 10.1113/jphysiol.1996.sp021152 8821137 PMC1158677

[B63] Pan Z. Z. Williams J. T. Osborne P. B. (1990). Opioid actions on single nucleus raphe magnus neurons from rat and guinea? pig in vitro. *J. Physiol.* 427 519–532. 10.1113/jphysiol.1990.sp018185 1976803 PMC1189944

[B64] Park C. Kim J.-H. Yoon B.-E. Choi E.-J. Lee C. J. Shin H.-S. (2010). T-type channels control the opioidergic descending analgesia at the low threshold-spiking GABAergic neurons in the periaqueductal gray. *Proc. Natl. Acad. Sci. U.S.A.* 107 14857–14862. 10.1073/pnas.1009532107 20682748 PMC2930412

[B65] Price D. D. Mayer D. J. Mao J. Caruso F. S. (2000). NMDA-receptor antagonists and opioid receptor interactions as related to analgesia and tolerance. *J. Pain Symptom Manage.* 19(1 Suppl.), S7–S11. 10.1016/S0885-3924(99)00121-9 10687332

[B66] Raghavendra V. Rutkowski M. D. DeLeo J. A. (2002). The role of spinal neuroimmune activation in morphine tolerance/hyperalgesia in neuropathic and sham-operated rats. *J. Neurosci.* 22 9980–9989. 10.1523/jneurosci.1850-04.2004 12427855 PMC6757841

[B67] Raghavendra V. Tanga F. Y. DeLeo J. A. (2004). Attenuation of morphine tolerance, withdrawal-induced hyperalgesia, and associated spinal inflammtory immune responses by propentofylline in rats. *Neuropsychopharmacology* 29 327–334. 10.1038/sj.npp.1300315 14532913

[B68] Ray S. B. Gupta H. Gupta Y. K. (2004). Up-regulation of mu-opioid receptors in the spinal cord of morphine-tolerant rats. *J. Biosci.* 29 51–56. 10.1007/BF02702561 15286403

[B69] Rönnbäck L. Hansson E. (1988). Are astroglial cells involved in morphine tolerance? *Neurochem. Res.* 13 87–103. 3283589 10.1007/BF00973320

[B70] Samineni V. K. Grajales-Reyes J. G. Copits B. A. O’Brien D. E. Trigg S. L. Gomez A. M. (2017). Divergent modulation of nociception by glutamatergic and gabaergic neuronal subpopulations in the periaqueductal gray. *Eneuro* 4:ENEURO.0129-16.2017. 10.1523/ENEURO.0129-16.2017 28374016 PMC5370278

[B71] Sharma S. K. Klee W. A. Nirenberg M. (1975). Dual regulation of adenylate cyclase accounts for narcotic dependence and tolerance. *Proc. Natl. Acad. Sci. U.S.A.* 72 3092–3096. 10.1073/pnas.72.8.3092 1059094 PMC432926

[B72] Smith F. L. Javed R. R. Smith P. A. Dewey W. L. Gabra B. H. (2006). PKC and PKA inhibitors reinstate morphine-induced behaviors in morphine tolerant mice. *Pharmacol. Res.* 54 474–480. 10.1016/j.phrs.2006.09.007 17056270

[B73] Song P. Zhao Z.-Q. (2001). The involvement of glial cells in the development of morphine tolerance. *Neurosci. Res.* 39 281–286. 10.1016/S0168-0102(00)00226-1 11248367

[B74] Stellwagen D. (2005). Differential Regulation of AMPA Receptor and GABA receptor trafficking by tumor necrosis factor-. *J. Neurosci.* 25 3219–3228. 10.1523/JNEUROSCI.4486-04.2005 15788779 PMC6725093

[B75] Stiller C. O. Bergquist J. Beck O. Ekman R. Brodin E. (1996). Local administration of morphine decreases the extracellular level of GABA in the periaqueductal gray matter of freely moving rats. *Neurosci. Lett.* 209 165–168. 10.1016/0304-3940(96)12638-0 8736636

[B76] Thomas J. Mustafa S. Johnson J. Nicotra L. Hutchinson M. (2015). The relationship between opioids and immune signaling in the spinal cord. *Handb. Exp. Pharmacol.* 227 207–238. 10.1007/978-3-662-46450-2_11 25846621

[B77] Torrecilla M. Marker C. L. Cintora S. C. Stoffel M. Williams J. T. Wickman K. (2002). G-protein-gated potassium channels containing Kir3.2 and Kir3.3 subunits mediate the acute inhibitory effects of opioids on locus ceruleus neurons. *J. Neurosci.* 22 4328–4334. 10.1523/JNEUROSCI.22-11-04328.2002 12040038 PMC6758804

[B78] Tortorici V. Morgan M. M. Vanegas H. (2001). Tolerance to repeated microinjection of morphine into the periaqueductal gray is associated with changes in the behavior of off- and on-cells in the rostral ventromedial medulla of rats. *Pain* 89 237–244. 10.1016/S0304-3959(00)00367-5 11166480

[B79] Vanderah T. W. Suenaga N. M. Ossipov M. H. Malan T. P. Lai J. Porreca F. (2001). Tonic descending facilitation from the rostral ventromedial medulla mediates opioid-induced abnormal pain and antinociceptive tolerance. *J. Neurosci.* 21 279–286. 10.1523/JNEUROSCI.21-01-00279.2001 11150345 PMC6762454

[B80] Vaughan C. W. Ingram S. L. Connor M. A. Christie M. J. (1997). How opioids inhibit GABA-mediated neurotransmission. *Nature* 390 611–614. 10.1038/37610 9403690

[B81] Viviani B. Bartesaghi S. Gardoni F. Vezzani A. Behrens M. M. Bartfai T. (2003). Interleukin-1β enhances NMDA receptor-mediated intracellular calcium increase through activation of the Src family of kinases. *J. Neurosci.* 23 8692–8700. 10.1523/JNEUROSCI.23-25-08692.2003 14507968 PMC6740426

[B82] Wang D. Zeng J. Li Q. Huang J. Couture R. Hong Y. (2016). Contribution of adrenomedullin to the switch of G protein-coupled μ-opioid receptors from Gi to Gs in the spinal dorsal horn following chronic morphine exposure in rats. *Br. J. Pharmacol.* 173 1196–1207. 10.1111/bph.13419 26750148 PMC5341334

[B83] Wang X. Loram L. C. Ramos K. de Jesus A. J. Thomas J. Cheng K. (2012). Morphine activates neuroinflammation in a manner parallel to endotoxin. *Proc. Natl. Acad. Sci. U.S.A.* 109 6325–6330. 10.1073/pnas.1200130109 22474354 PMC3341002

[B84] Watkins L. R. Hutchinson M. R. Rice K. C. Maier S. F. (2009). The “toll” of opioid-induced glial activation: improving the clinical efficacy of opioids by targeting glia. *Trends Pharmacol. Sci.* 30 581–591. 10.1016/j.tips.2009.08.002 19762094 PMC2783351

[B85] Wilding T. J. Womack M. D. McCleskey E. W. (1995). Fast, local signal transduction between the mu opioid receptor and Ca2+ channels. *J. Neurosci.* 15(5 Pt 2), 4124–4132. 10.1523/JNEUROSCI.15-05-04124.1995 7538571 PMC6578243

[B86] Williams J. T. Christie M. J. Manzoni O. (2001). Cellular and synaptic adaptations mediating opioid dependence. *Physiol. Rev.* 81 299–343. 10.1152/physrev.2001.81.1.299 11152760

[B87] Wimpey T. L. Chavkin C. (1991). Opioids activate both an inward rectifier and a novel voltage-gated potassium conductance in the hippocampal formation. *Neuron* 6 281–289. 10.1016/0896-6273(91)90363-5 1993123

[B88] Xiao Z. Li Y. Y. Sun M. J. (2015). Activation of P2X7 receptors in the midbrain periaqueductal gray of rats facilitates morphine tolerance. *Pharmacol. Biochem. Behav.* 135 145–153. 10.1016/j.pbb.2015.06.002 26054441

[B89] Xie J. Y. Herman D. S. Stiller C.-O. Gardell L. R. Ossipov M. H. Lai J. (2005). Cholecystokinin in the rostral ventromedial medulla mediates opioid-induced hyperalgesia and antinociceptive tolerance. *J. Neurosci.* 25 409–416. 10.1523/JNEUROSCI.4054-04.2005 15647484 PMC6725495

[B90] Xu J. Sun L. Lutz B. Bekker A. T. Tao Y. (2015). Intrathecal rapamycin attenuates morphine-induced analgesic tolerance and hyperalgesia in rats with neuropathic pain. *Transl. Perioper. Pain Med.* 2 27–34. 26339682 PMC4556423

[B91] Xu J.-T. T. Yaster M. Tao Y.-X. X. Zhao J.-Y. Y. Zhao X. Ligons D. (2014). Opioid receptor-triggered spinal mTORC1 activation contributes to morphine tolerance and hyperalgesia. *J. Clin. Invest* 124 592–603. 10.1172/JCI70236 24382350 PMC3904613

[B92] Zhao Y. L. Chen S. R. Chen H. Pan H. L. (2012). Chronic opioid potentiates presynaptic but impairs postsynaptic N-methyl-D-aspartic acid receptor activity in spinal cords: implications for opioid hyperalgesia and tolerance. *J. Biol. Chem.* 287 25073–25085. 10.1074/jbc.M112.378737 22679016 PMC3408179

[B93] Zhou D. Chen M.-L. Zhang Y.-Q. Zhao Z.-Q. (2010). Involvement of Spinal microglial P2X7 receptor in generation of tolerance to morphine analgesia in rats. *J. Neurosci.* 30 8042–8047. 10.1523/JNEUROSCI.5377-09.2010 20534852 PMC6632682

